# Cu-HEPES Nanosheets with Dual Enzyme-Mimetic Activities for Colorimetric Detection of 2,4-Dichlorophenol

**DOI:** 10.3390/molecules31152639

**Published:** 2026-07-29

**Authors:** Jia-Yuan He, Hao Zhang

**Affiliations:** 1 Traditional Chinese Medicine College, Chongqing University of Chinese Medicine, Chongqing 402760, China; 2 Chongqing Engineering Research Center of Pharmaceutical Sciences, Chongqing Medical and Pharmaceutical College, Chongqing 401331, China

**Keywords:** Cu-HEPES nanosheets, laccase-mimicking nanozyme, oxidase-mimicking nanozyme, 2,4-dichlorophenol detection, dye removal

## Abstract

A convenient one-pot strategy was adopted to fabricate Cu-4-(2-hydroxyethyl)-1-piperazineethanesulfonic acid (HEPES) nanosheets through the coordination of HEPES with copper ions under alkaline conditions, and the obtained nanosheets possessed dual laccase-mimetic and oxidase-mimetic activities for phenolic pollutant analysis and show potential for dye decolorization/removal. The Cu-HEPES nanosheets displayed significant dual enzyme-mimetic activities, effectively catalyzing the oxidation conversion for phenolic compounds, specifically 2,4-dichlorophenol (2,4-DCP), to yield quinone imine products characterized by a unique absorption signal at 500 nm. Meanwhile, Cu-HEPES nanosheets also facilitated the conversion of 3,3′,5,5′-tetramethylbenzidine to yield a yellow-green substance with a distinctive absorption signal at 458 nm. The Cu-HEPES nanosheets displayed a satisfactory affinity (*K*_m_ = 23.2 μM) for 2,4-DCP, facilitating its use in accurately detecting 2,4-DCP (linear range from 3.3 to 16.6 µM) with a detection limit of 0.3 µM. Subsequently, the devised colorimetric sensing platform was applied to measure 2,4-DCP levels in real tap water samples, yielding satisfactory spiked recoveries ranging from 92.7% to 106.9%. Finally, the synthesized Cu-HEPES nanosheets were preliminarily evaluated for the simultaneous decolorization/removal of malachite green and Congo red, suggesting their potential applicability in aqueous systems containing multiple organic dyes.

## 1. Introduction

The rapid-paced progress of industrialization in recent years has brought about different levels of impairment to the ecological system, most notably the water resource environment [1,2]. Phenolic substances and colorants, common pollutants in water bodies, have become major pollutants owing to their widespread use in plastics, agriculture, dyeing, and medicinal products [3,4]. Even tiny amounts of these pollutants discharged into water can inflict serious damage on animals, plants, aquatic life, and human well-being. These phenolic compounds are believed to pose a threat to human health, with potential harms including carcinogenicity, reproductive toxicity, neurotoxicity, and endocrine disruption [5,6]. In addition, the accumulation of phenolic compounds in the human body through long-term metabolism can pose a threat to health. Accordingly, the efficient monitoring and elimination of organic pollutants from wastewater have garnered substantial academic interest.

Numerous techniques are widely adopted to remove industrial organic pollutants, including membrane filtration [7], coagulation [8], catalytic oxidation [9,10], and adsorption [11]. Each of these methods possesses distinct characteristics and applications in environmental remediation. Membrane filtration operates through physical separation mechanisms, effectively removing suspended solids and macromolecular contaminants. Coagulation involves the addition of chemical agents to neutralize colloidal particles, facilitating their aggregation and subsequent removal. Adsorption relies on the surface affinity of adsorbent materials to capture and retain pollutant molecules.

Among these approaches, catalytic oxidation is notable as a highly efficient strategy for breaking down organic contaminant compounds, featuring high efficiency, affordability, and little environmental disturbance. This approach boosts water quality and wastewater treatment efficiency via thorough oxidation of organic contaminants into non-toxic final byproducts, primarily carbon dioxide and water [12,13]. This oxidative process can transform organic contaminants into less harmful products under suitable conditions, rather than simply transferring them from one phase to another. On the other hand, catalytic oxidation demonstrates remarkable versatility in treating various categories of organic contaminants, including persistent organic pollutants, dyes, pharmaceuticals, and industrial chemicals. Natural enzymes have been extensively applied in diverse fields. For instance, Yuan et al. [14] demonstrated the immobilization of natural laccase within a metal–organic framework to collectively boost dye oxidation reactions. However, free laccase exhibited a sharp decline in stability when subjected to acid or base conditions, highlighting the susceptibility of natural enzymes to environmental factors. Moreover, natural enzymes possess inherent shortcomings like inadequate stability, elevated costs, and challenges with reusability, which hamper their large-scale practical application.

Latest progress in nanomaterial technology has unveiled a distinct category of nanomaterials exhibiting catalytic properties similar to natural enzymes. These innovative nanostructures, commonly referred to as nanozymes, represent a significant breakthrough in the field of nanotechnology and biochemistry [15,16]. Nanozymes exhibit superior characteristics compared to natural enzymes, including excellent stability, robust catalytic performance, and adjustable enzyme-mimetic activity [16,17]. Various nanozymes simulate natural enzyme catalytic behaviors, including laccase-mimetic, superoxide-dismutase-mimetic, oxidase-mimetic, peroxidase-mimetic, and hydrolase-mimetic activities [18]. Among these, laccase is particularly characterized by possessing a catalytically active site that comprises four copper ions strategically positioned within its three-dimensional protein structure. These copper ions work in concert to facilitate the significant acceleration of substrate oxidation reactions. Specifically, laccase demonstrates catalytic efficiency toward a diverse range of organic substrates, including phenolic compounds, polyphenolic substances with multiple hydroxyl groups, and various amine-containing molecules. The catalytic mechanism operates through a sophisticated electron transfer process, wherein the copper ions sequentially accept electrons from the substrate molecules, subsequently transferring these electrons to molecular oxygen as the final electron acceptor, thereby generating water as a byproduct. This electron transfer pathway not only enables the rapid oxidation of these substrates but also contributes to the enzyme’s remarkable catalytic versatility and substrate specificity, making laccase an invaluable biocatalyst in numerous industrial and environmental applications [19]. After conducting an in-depth investigation into the catalytic mechanism of laccase, multiple copper-based nanozymes have been fabricated to simulate the catalytic function of this enzyme. For instance, Han et al. [20] put forward a flexible approach for the synthesis of two laccase-mimetic nanozymes, which were utilized for the identification of eleven halogenated phenolic pollutants. Previously, our group [21] developed a nanozyme assembled from 2-methylimidazole and copper ions, which displayed laccase-mimetic activity and was employed for the decontamination of phenolic contaminants. The nanozyme mentioned above demonstrated the capacity to remove particular pollutants from wastewater without the need for light or additional conditions. However, it is constrained by low catalytic efficiency and limited enzyme-mimicking activities, highlighting the urgent need for developing a novel nanozyme that combines high catalytic performance with a variety of enzyme-mimetic activities.

**Figure 1:**
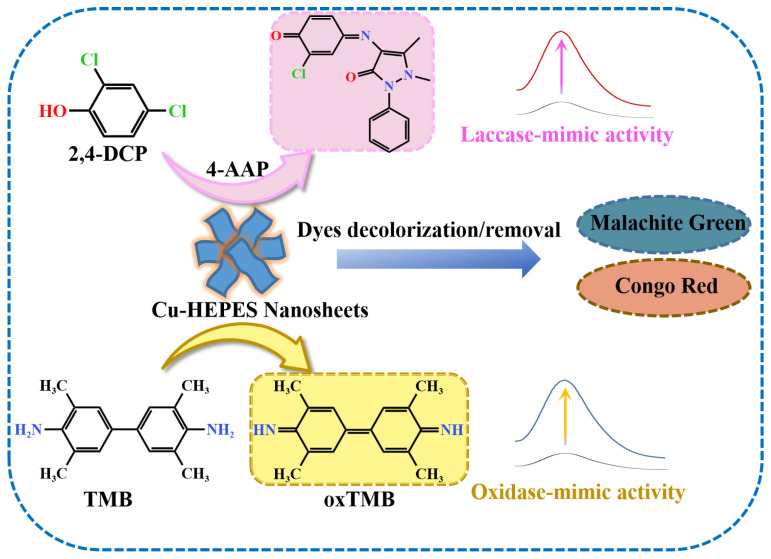
Schematic illustration of the green synthesis of Cu-HEPES nanosheets and their laccase- and oxidase-mimetic applications.

Different from conventional imidazole- or carboxylate-based ligands, 4-(2-hydroxyethyl)-1-piperazineethanesulfonic acid (HEPES) contains multiple functional groups, including piperazine nitrogen atoms, hydroxyl groups, and sulfonate groups. These groups can participate in the coordination with Cu^2+^ and contribute to the formation of Cu-HEPES nanosheets under mild alkaline aqueous conditions. Therefore, HEPES acts not only as a ligand but also as a structure-directing component for the rapid and convenient construction of Cu-based nanosheets. In this study, the synthesis of Cu-HEPES nanosheets with dual enzyme-mimetic activities (oxidase- and laccase-mimetic) was achieved by coordinating HEPES with copper ions under an alkaline environment for the detection of phenolic compounds and removal of dyes (Figure 1). The synthesized Cu-HEPES nanosheets exhibit dual enzyme-mimetic catalytic functions, driving the reaction between 2,4-dichlorophenol (2,4-DCP) and 4-aminoantipyrine (4-AAP) to generate a quinone imine (λ_max_ = 500 nm), as well as oxidizing 3,3′,5,5′-tetramethylbenzidine (TMB) to form a yellow-green product (λ_max_ = 458 nm). This study further investigated the substrate selectivity and affinity of Cu-HEPES nanosheets and systematically examined the influences of pH value, reaction temperature, and reaction duration on their relative catalytic activity (%). Finally, the Cu-HEPES nanosheets were employed for the detection of 2,4-DCP in real samples and were preliminarily evaluated for the simultaneous decolorization/removal of malachite green and Congo red, indicating their potential for further application in aqueous systems containing phenolic pollutants and multiple dyes. Unlike previously reported copper-based laccase-like nanozymes, HEPES was selected here not only as a mild coordinating ligand but also as a multifunctional structure-directing component and provides a simple route to Cu-HEPES nanosheets with dual laccase-like and oxidase-like activities. The main contribution of this work is the HEPES-assisted construction of Cu-HEPES nanosheets and the demonstration of their bifunctional enzyme-mimetic catalytic behavior. The obtained Cu-HEPES nanosheets exhibit both laccase-mimetic activity toward 2,4-DCP/4-AAP oxidation and oxidase-mimetic activity toward TMB oxidation. Therefore, this work demonstrates the potential application of Cu-HEPES nanosheets in colorimetric phenolic pollutant detection and in preliminary tests of dye decolorization/removal.

## 2. Results and Discussion

### 2.1. Characterization

**Figure 2:**
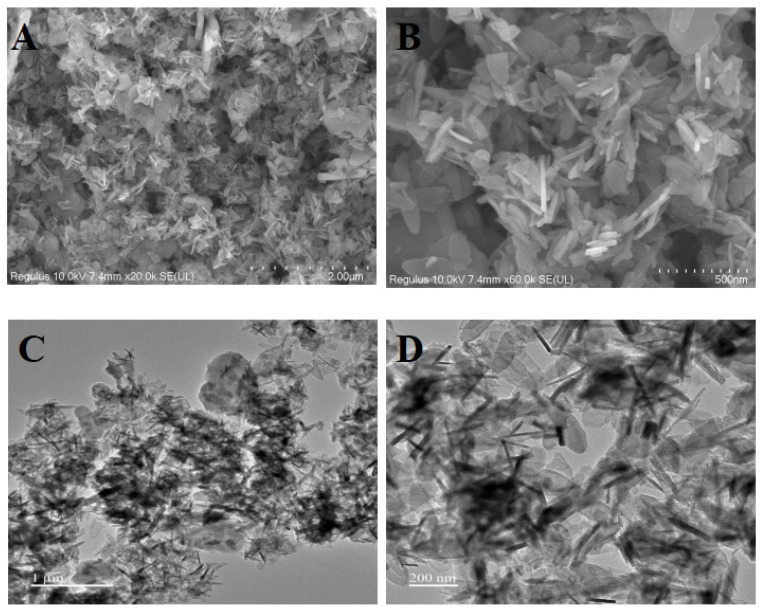
SEM (**A**,**B**) and TEM (**C**,**D**) images of the Cu-HEPES nanosheets.

**Figure 3:**
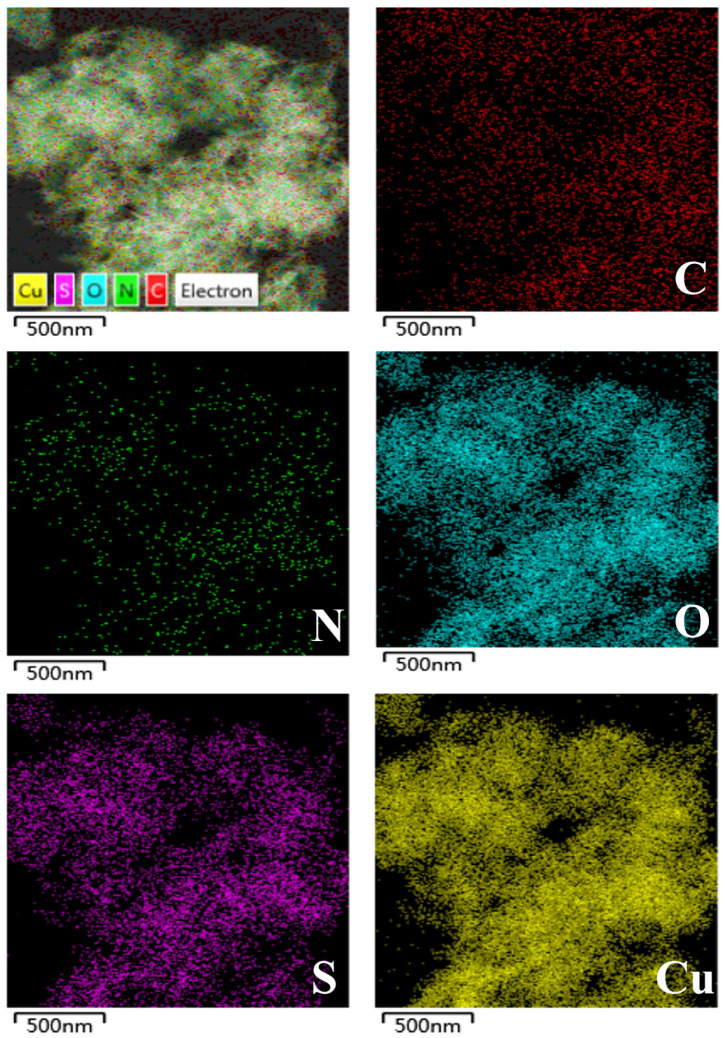
EDS elemental mapping images of Cu-HEPES nanosheets.

The synthesized Cu-HEPES material was systematically analyzed using various characterization techniques, with morphological features obtained through observation using SEM. SEM images of Cu-HEPES material (Figure 2A,B) reveal irregular sheet-shaped nanostructures, indicating successful synthesis of Cu-HEPES nanosheets. TEM characterization results (Figure 2C,D) further support this sheet-shaped nanostructure. We also collected EDS elemental mapping images for the synthesized Cu-HEPES nanosheets, and the obtained results demonstrate that C, N, O, S, and Cu elements are all distributed on their surface (Figure 3).

**Figure 4:**
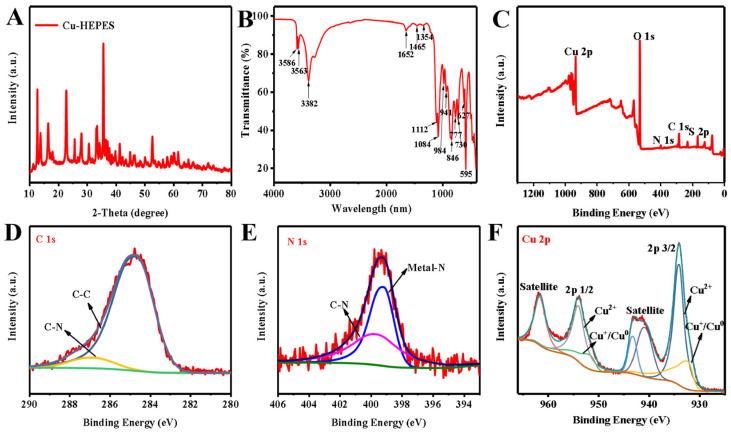
XRD (**A**) and FT-IR (**B**) images of the Cu-HEPES nanosheets. XPS analysis of the Cu-HEPES nanosheets: element survey (**C**); C 1s (**D**); N 1s (**E**); Cu 2p (**F**).

As shown in Figure 4A, the XRD pattern of Cu-HEPES nanosheets exhibits several sharp diffraction peaks at 2*θ* = 12.74°, 13.82°, 16.5°, 25.66°, 28.02°, 30.60°, 33.44°, 34.3°, 35.64°, 36.48°, 37.08°, 41.28°, and 52.62°. These well-defined diffraction peaks confirm the crystalline nature of the synthesized Cu-HEPES nanosheets. Additionally, the sharp and intense diffraction peaks indicate the ordered coordination assembly between Cu^2+^ and HEPES, supporting the successful formation of crystalline Cu-HEPES nanosheets. Figure 4B shows the FT-IR spectral profiles collected from the synthesized Cu-HEPES nanosheets, where clear spectral signals are detected at 3586, 3563, and 3382 cm^−1^ across the 3300–3600 cm^−1^ wavenumber interval. These peaks are attributed to the O–H bond stretching modes belonging to the hydroxyethyl group (–CH_2_CH_2_OH) present in the HEPES compound. Notably, the absorption signal located at 3586 cm^−1^ is ascribed to free hydroxyl groups. In comparison, the distinct spectral bands located at 3563 cm^−1^ and 3382 cm^−1^ originate from weakly and strongly associated hydroxyl groups, respectively. The peak broadening and red shift indicate that the hydroxyl oxygen atoms participate in the coordination with Cu^2+^, and strong hydrogen bonds exist between molecules. In the 1300–1700 cm^−1^ region, the strong absorption signals at 1652 cm^−1^ and 1354 cm^−1^ correspond to asymmetric and symmetric stretching vibrational modes of the S=O in the sulfonate group (–SO_3_^−^), respectively, confirming the integrity of the sulfonate structure in the HEPES ligand. A distinct infrared band at 1465 cm^−1^ originates from the coupling of the C–N tensile vibration of the piperazine ring and the flexural vibration of the methylene molecular moiety (–CH_2_–), reflecting the characteristic vibrational mode of the heterocyclic skeleton. The infrared bands at 1112, 1084, 984, and 941 cm^−1^ correspond to the stretching vibrational modes of C–O bonds from the hydroxyethyl moiety and C–N bonds within the piperazine ring, verifying the presence of the hydroxyethyl side chain. The signals centered at 846, 777, and 730 cm^−1^ are assigned to the skeletal oscillation behaviors, as well as the out-of-plane bending vibrations of the alkyl chain and piperazine ring. Notably, the signal observed at 627 and 595 cm^−1^ arises from the characteristic vibrations associated with the coordination bonds formed between Cu^2+^ and N (piperazine ring) and O (hydroxyl/sulfonate) atoms in HEPES, directly proving the successful coordination of the Cu-HEPES complex. In summary, FT-IR analysis reveals that the functional groups of HEPES are fully retained in the complex, and Cu^2+^ is loaded onto the ligand via coordination with N and O atoms, forming a stable Cu-HEPES metal–organic complex. The XPS spectrum in Figure 4C shows the presence of C, N, O, S, and Cu elements, confirming the successful formation of Cu-HEPES nanosheets. The additional features observed around 570 and 650 eV are associated with Cu LMM Auger-related signals in the survey scan. From the C 1s high-resolution scan data presented in Figure 4D, signal peak attribution for the feature with a binding energy of 284.6 eV aligns with C–C chemical linkages, while the 286.8 eV feature matches C–N chemical linkages. Furthermore, Figure 4E illustrates a high-resolution XPS N 1s profile showing two prominent characteristic signals at 399.1 eV and 399.6 eV, representing metal–N and C–N, respectively. This highlights the role of N in stabilizing Cu atoms within the nanozyme structure. In addition, two satellite peaks are also observed, signifying the existence of Cu^2+^ species [22]. The characteristic signals of Cu^2+^ appear as two spectral features at 934.0 eV (Cu 2p_3/2_) and 954.0 eV (Cu 2p_1/2_) in the high-resolution Cu 2p spectrum (Figure 4F) [23]. These results demonstrate that the copper element in the Cu-HEPES nanosheet exists primarily as Cu^2+^. Peaks appearing at 932.3 and 951.7 eV can be attributed to Cu^+^ and/or Cu^0^, as their binding energies in the XPS spectrum are nearly identical [22]. These results suggest the presence of Cu species with different chemical environments in the Cu-HEPES nanosheets. Based on the FT-IR and XPS analyses, a possible formation mechanism of Cu-HEPES nanosheets can be proposed. Under alkaline conditions, the N atoms in the piperazine ring and the O-containing groups of HEPES can coordinate with Cu^2+^, leading to the rapid formation of Cu-HEPES coordination networks. Meanwhile, the sulfonate and hydroxyl groups of HEPES may improve the dispersion of the coordination units in aqueous solution and guide their anisotropic assembly into sheet-like nanostructures. Therefore, HEPES serves not only as a ligand for Cu^2+^ coordination but also as a structure-directing component for nanosheet formation.

### 2.2. Analysis of the Laccase-Mimetic and Oxidase-Mimetic Activities Exhibited by Cu-HEPES Nanosheets

**Figure 5:**
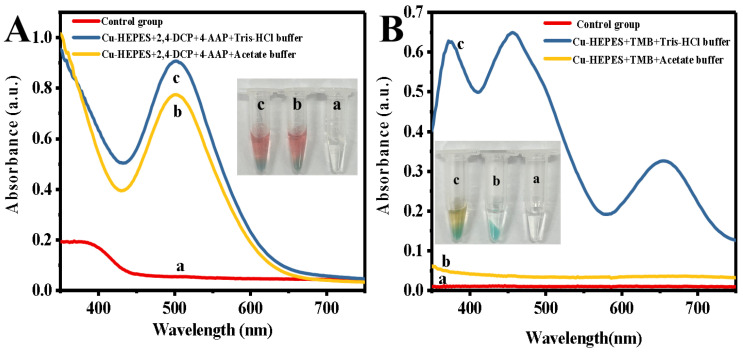
(**A**) UV–vis absorption spectra of a mixture of Cu-HEPES nanosheets, 4-AAP, 2,4-DCP, Tris-HCl buffer or acetate buffer solution (insets: the corresponding images); (**B**) UV–vis absorption spectra of a mixture of Cu-HEPES nanosheets, TMB, Tris-HCl buffer or acetate buffer solution (insets: the corresponding images).

This study systematically investigates the dual enzyme-mimetic activity of Cu-HEPES to broaden its applications in the field of biosensors. Cu-HEPES nanosheets had phenol oxidation and redox catalytic traits gauged via well-established color-developing assays, with 2,4-DCP and TMB as color-developing substrates for each trait separately. In Figure 5A, the blend of 2,4-DCP alongside 4-AAP within aqueous media did not yield a noticeable color change (curve a). Conversely, the presence of Cu-HEPES nanosheets caused a spectroscopic signal centered at 500 nm (curves b and c), confirming its laccase-mimetic activity. The color alteration in the solution is visually observable, as presented in the inset of Figure 5A. Importantly, the catalytic efficiency of Cu-HEPES nanosheets exhibits a notable dependence on the buffer system, with optimal performance observed in Tris-HCl buffer (curve c), while the activity is relatively lower in acetate buffer (curve b).

Figure 5B illustrates the catalytic activity of Cu-HEPES nanosheets as oxidase mimics. In the presence of only TMB solution, no significant color change is observed (inset in Figure 5B). Conversely, the incorporation of Cu-HEPES nanosheets into the Tris-HCl buffer produces a prominent absorption peak at 458 nm (curve c) and yields a yellow-green solution (inset in Figure 5B), thus verifying the remarkable oxidase-mimetic capability of Cu-HEPES nanosheets. Similarly, the oxidase-mimetic catalytic activity of Cu-HEPES nanosheets also exhibits a notable dependence on the buffer system, with optimal performance observed in Tris-HCl buffer (curve c), while exhibiting almost no activity in acetate buffer (curve b). The buffer-dependent oxidase-mimetic activity may be related to the different coordination environments provided by Tris-HCl and acetate buffers. Tris contains both amino and hydroxyl groups, which can chelate with Cu ions and may help stabilize or regulate the coordination microenvironment of Cu-based catalytic sites, thereby facilitating electron transfer and TMB oxidation. In contrast, acetate cannot provide a similar chelating environment for Cu ions under the present conditions, which may result in much weaker oxidase-mimetic activity. Therefore, Cu-HEPES nanosheets showed much higher catalytic activity in Tris-HCl buffer than in acetate buffer. Finally, Tris-HCl buffer was used in the next study. This bifunctional catalytic behavior distinguishes Cu-HEPES nanosheets from our previously reported Cu-MOF nanosheets [21], which primarily focused on laccase-mimetic catalysis. The introduction of HEPES as a multifunctional ligand may contribute to the formation of accessible Cu-based catalytic sites, thereby favoring the coexistence of laccase-mimetic and oxidase-mimetic activities. The dual enzyme-mimetic activity of Cu-HEPES nanosheets may be related to the accessible Cu-based catalytic sites stabilized by the multifunctional HEPES ligand. In the laccase-mimetic reaction, these Cu sites may promote electron transfer from 2,4-DCP/4-AAP to dissolved oxygen, leading to the formation of quinone imine products with a characteristic absorption peak at 500 nm. In the oxidase-mimetic reaction, the Cu-based sites may facilitate the activation of dissolved oxygen and accelerate TMB oxidation, producing a yellow-green oxidized product with an absorption peak at 458 nm. These results suggest that the HEPES-assisted coordination structure is favorable for bifunctional enzyme-mimetic catalysis.

### 2.3. Laccase-Mimetic Activity of Cu-HEPES Nanosheets

**Figure 6:**
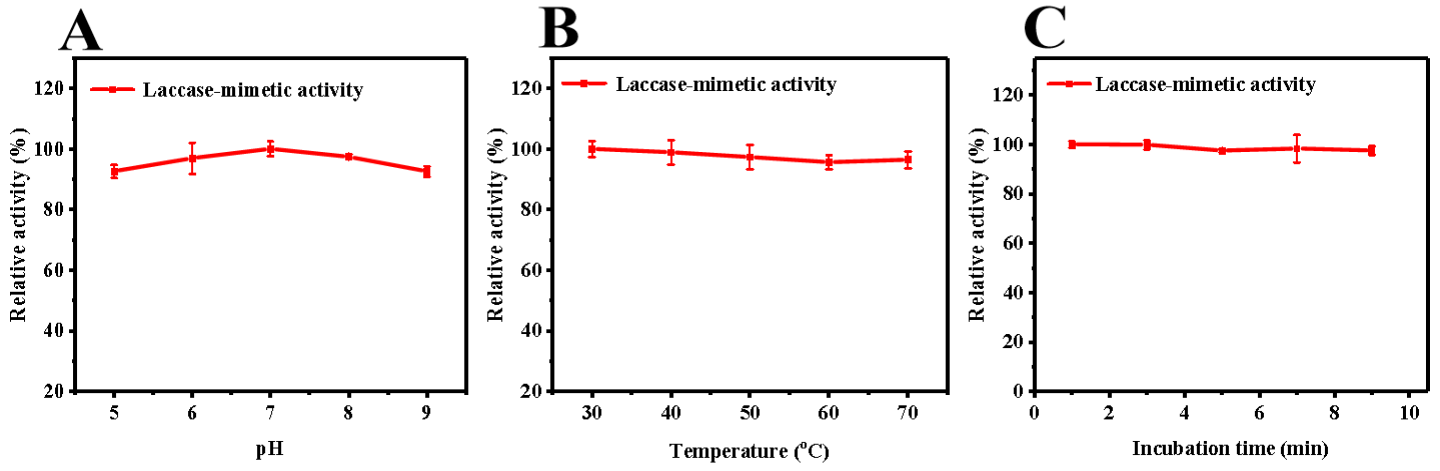
Effect of pH (**A**), temperature (**B**), and incubation time (**C**) on the relative laccase-mimetic activity of the Cu-HEPES nanosheets. Conditions: temperature = 37 °C, incubation time = 1.0 min for (**A**); pH = 7.0, incubation time = 1.0 min for (**B**); and pH = 7.0, temperature = 30 °C for (**C**).

Given that pH, temperature, and incubation time have been shown to influence the catalytic activity of laccase, this study systematically evaluated their effects on the relative catalytic performance (%) of Cu-HEPES nanosheets. Figure 6A illustrates that the relative catalytic performance (%) of Cu-HEPES nanosheets enhances with increasing pH in the Tris-HCl buffer system. However, there is a slight reduction in relative catalytic activity (%) once the pH exceeds 7.0. Hence, Cu-HEPES nanosheets are deemed suitable for catalytic reactions under neutral conditions, and pH 7.0 was selected for all follow-up experiments. Subsequently, the influence of enzymatic reaction temperature on relative activity (%) was investigated. In Figure 6B, Cu-HEPES nanosheets exhibit high thermal stability between 30 °C and 70 °C, with only a slight 5% decrease in relative catalytic activity. Hence, considering the balance between catalytic performance and experimental feasibility, all subsequent reactions were performed at 30 °C. Ultimately, we investigated how incubation time ranging from 1.0 to 9.0 min affects the relative catalytic performance of the prepared nanosheet. The relative activity (%) of Cu-HEPES nanosheets shows minimal variation with prolonged enzyme reaction time, as depicted in Figure 6C, suggesting their excellent laccase-mimetic catalytic efficiency. Consequently, the catalytic reaction duration was fixed at 1.0 min for the subsequent experiments.

Apparent Michaelis–Menten parameter analysis was adopted with 2,4-DCP as the reaction substrate to evaluate the laccase-mimetic performance of as-prepared Cu-HEPES nanosheets. In Figure S1A, an increase in substrate concentration resulted in a corresponding elevation in absorbance value. The kinetic parameter was fitted to the Michaelis–Menten equation and subsequently converted into a Lineweaver–Burk double reciprocal plot. In Figure S1B, it demonstrates an excellent linear fitting relationship with the equation Y = 21.215X + 0.9151 (R^2^ = 0.9913). The *K*_m_ value of 2,4-DCP for Cu-HEPES nanosheets is 23.2 ± 6.4 μM, significantly lower than other similar laccase-mimetic nanozymes listed in Table S1, indicating its excellent affinity for this substrate.

### 2.4. Colorimetric Detection of 2,4-DCP Using Cu-HEPES Nanosheets

**Figure 7:**
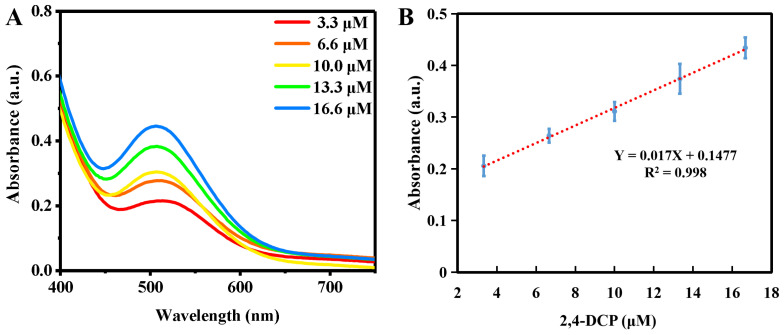
(**A**) UV–vis absorption spectra of 4-AAP incubated with the Cu-HEPES nanosheets in the presence of varying concentrations of 2,4-DCP; (**B**) the linear relationship between 2,4-DCP concentration and the corresponding absorption intensity at 500 nm.

The absorbance values exhibit a gradual increase with the rise in 2,4-DCP concentration from 3.3 to 16.6 µM, as illustrated in Figure 7A. The calibration plot displayed in Figure 7B was constructed by correlating 2,4-DCP concentrations with optical absorbance readout recorded at 500 nm. The derived regression function was expressed as Y = 0.017X + 0.1477, in which X refers to 2,4-DCP concentration and Y stands for the optical signal at 500 nm, yielding a high R^2^ value of 0.998. This low-concentration range was selected as the quantitative linear range because it provided stable and sensitive absorbance responses suitable for sensitive detection of trace 2,4-DCP under the optimized assay conditions. By applying the classical 3*σ*/*S* calculation method, where *σ* stands for standard deviation and *S* refers to the calibration curve slope, the limit of detection (LOD) was determined to be 0.3 µM. In Table 1, the colorimetric assay achieves a more favorable LOD compared to other reported methods for 2,4-DCP analysis.

Moreover, this study assessed the analytical capability and detection accuracy of the developed method for 2,4-DCP by selectively testing actual samples. The selected interfering substances, such as Na^+^, Mg^2+^, Ca^2+^, CO_3_^2−^, Fe^3+^, and Zn^2+^, are components that may be present in real samples. The concentration of 2,4-DCP is 0.01 mM, while that of the interfering substances is 0.02 mM. During the selectivity study, interfering substances were introduced into the enzymatic reaction mixture in place of 2,4-DCP. The analysis depicted in Figure S2A reveals that there were no notable alterations in the absorbance value for the interfering substances. The interfering substances were individually mixed with 2,4-DCP for the interference study and subsequently analyzed using the UV–vis spectrophotometer. Figure S2B demonstrates the absence of significant changes in the absorbance value when exposed to interfering substances. These findings suggest that the current technique for 2,4-DCP detection exhibits excellent selectivity and anti-interference capability.

Finally, the current method relies on Cu-HEPES nanosheets to mimic laccase-mimetic activity and assess their practical detection ability for 2,4-DCP. The spike recovery rates for 6.6, 10.0, and 13.3 μM of 2,4-DCP added to two water samples and the corresponding recovery outcomes are presented in Table 2. All tested concentration levels yielded recovery rate between 92.7% and 106.9% in both samples, demonstrating the accuracy of the developed colorimetric detection method for 2,4-DCP. Some recovery rates in Table 2 were slightly higher than 100% because the measured concentrations after spiking were marginally higher than the theoretical spiked concentrations. Since the recovery rate was calculated as the ratio of the measured increase in concentration to the added concentration, even a small positive deviation in absorbance or calculated concentration can lead to recovery values above 100%. Such positive deviations may originate from the matrix effect of real water samples and normal experimental errors during sample spiking, dilution, and absorbance measurement. Nevertheless, the recovery rate remained within an acceptable range, indicating that the proposed method can be applied to 2,4-DCP detection in real water samples.

### 2.5. Oxidase-Mimetic Activity of Cu-HEPES Nanosheets

The relative oxidase-mimetic catalytic performance (%) of Cu-HEPES nanosheets was examined to assess the impact of pH, temperature, and incubation time, all of which are known to influence the catalytic capability of natural enzymes. In Figure S3A, the relative activity of Cu-HEPES nanosheets is optimal at pH 6.0. However, the relative catalytic activity decreases when the pH is below or above 6.0, with only 35.7% of the initial activity retained at pH 9.0. These findings indicate that its oxidase-mimetic catalytic function is most suitable under slightly acidic conditions. Subsequently, the impact of temperature (30–70 °C) on the relative oxidase-mimetic activity (%) was investigated. In Figure S3B, the relative oxidase-mimetic catalytic activity (%) of Cu-HEPES nanosheets declines markedly as temperature rises, indicating the susceptibility of Cu-HEPES nanosheets’ oxidase-mimetic catalytic activity to high temperatures. Finally, the impact of incubation time (1.0–9.0 min) on the relative oxidase-mimetic activity (%) was investigated. According to Figure S3C, the relative catalytic performance (%) of Cu-HEPES nanosheets declines gradually with prolonged reaction time, possibly due to the oxidation of the substrate TMB, forming distinct electron transfer complexes.

Apparent Michaelis–Menten parameter analysis was employed to characterize the oxidase-mimetic activity of Cu-HEPES nanosheets, using TMB as the substrate. As depicted in Figure S4A, as the substrate concentration increases, the absorbance value also rises accordingly. The apparent Michaelis–Menten parameter was determined by fitting it to the Michaelis–Menten equation, which was then transformed into a Lineweaver–Burk double reciprocal plot. This plot, shown in Figure S4B, exhibits a significant linear relationship described by the equation Y = 0.0734X + 0.1401 (R^2^ = 0.9799). Cu-HEPES nanosheets possess outstanding binding affinity toward TMB, as indicated by their low *K*_m_ of 0.52 ± 0.07 mM. Compared with previously reported other nanozymes with oxidase-mimetic properties listed in Table S2, the Cu-HEPES nanosheets exhibit a substantially lower Km value, highlighting their strong binding to TMB.

### 2.6. Dye Removal

**Figure 8:**
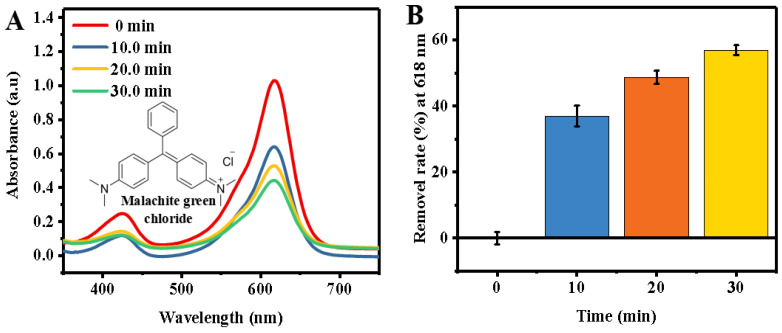
(**A**) UV–vis spectra of the malachite green solutions after incubation with Cu-HEPES nanosheets and H_2_O_2_; (**B**) removal rate (%) of malachite green calculated from the absorbance at 618 nm.

Figure 8A displays the time-resolved spectral signal of malachite green acquired at various intervals using Cu-HEPES nanosheets as a catalyst. With the catalytic reaction duration increasing continuously, the characteristic absorption signal at 618 nm showed a gradual decrease. This spectral variation clearly verifies the effective removal of malachite green. The removal efficiency of malachite green was 37% at 10.0 min, which increased to 49% at 20.0 min and ultimately reached 57% after 30.0 min, as shown in Figure 8B.

**Figure 9:**
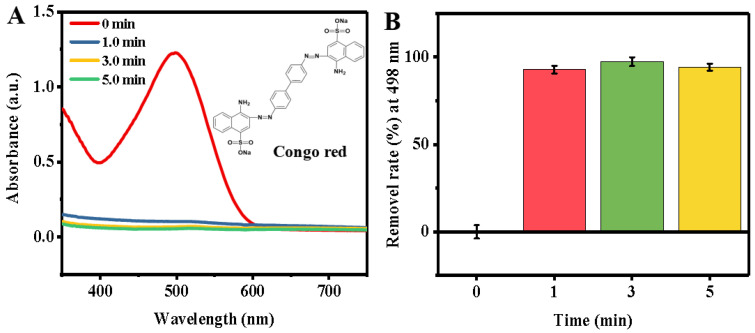
(**A**) UV–vis spectra of the Congo red solutions after incubation with Cu-HEPES nanosheets and H_2_O_2_; (**B**) removal rate (%) of Congo red calculated from the absorbance at 498 nm.

Similarly, spectral scanning of Congo red in the reaction system containing Cu-HEPES nanosheets was also conducted at various time points (Figure 9A). Following 1.0 min, the absorption peak of Congo red at 498 nm diminishes notably, yielding a calculated removal rate of 92% for Congo red. Subsequent extension of the incubation period maintains a nearly constant removal rate (Figure 9B). These outcomes suggest that Cu-HEPES nanosheets exhibit higher Congo red removal efficiency than malachite green. The synthesis of Cu-HEPES nanosheets in this study is simplified under mild conditions compared to prior studies [32,33,34,35], while showing similar or improved efficacy in pollutant elimination.

**Figure 10:**
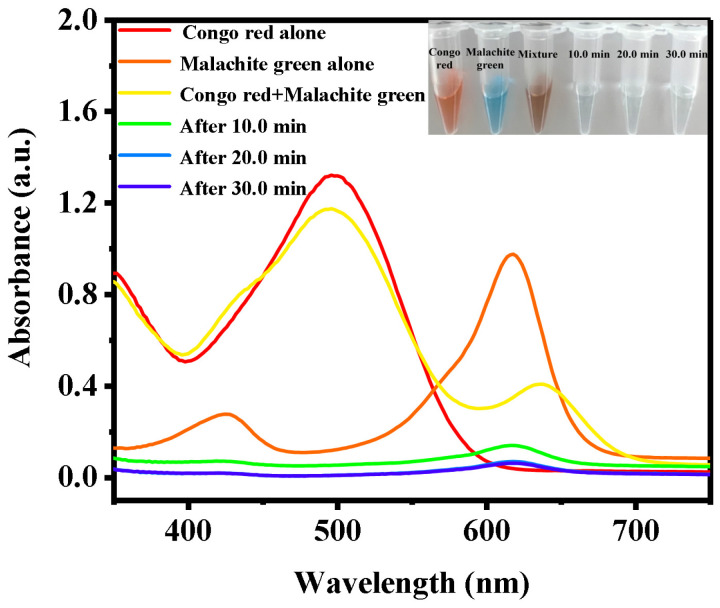
UV–vis spectra of the Cu-HEPES nanosheets for simultaneous removal of Congo red and malachite green (insets: the corresponding images).

On the other hand, real water contamination samples commonly contain various dyes from industrial processes, agricultural runoff, or household products, resulting in complex mixtures that are challenging to detect and remediate. Hence, an experiment was performed to remove Congo red and malachite green by mixing their anion/cation solutions. As illustrated in Figure 10, coexisting anionic and cationic dyes in mixed solutions induce variations in absorption peak intensity and wavelength position, which differ from the spectral characteristics of single-component dye systems [36]. During the removal experiment, the Cu-HEPES nanosheets completely removed Congo red within 10.0 min. Simultaneously, the absorption peak intensity of malachite green at 618 nm decreased steadily as the reaction time extended. Visual chromatic photographs of the corresponding solutions are also shown in Figure 8. On the other hand, the crystal structure of Cu-HEPES nanosheets was analyzed using XRD to study the effects of dye removal. As shown in Figure S5, all samples exhibit strong diffraction peaks, indicating high crystallinity. In the removal experiment of malachite green, the XRD of Cu-HEPES nanosheets before and after removal shows consistent peak positions with only slight variations in peak intensities, suggesting that the molecular arrangement remains largely unchanged during this process. Conversely, in the removal experiment of Congo red, while most diffraction peaks remain, some peaks disappear or exhibit significantly reduced intensities, indicating a marked alteration in molecular arrangement. These differences may stem from the distinct removal mechanisms of these two dyes. It should be noted that the dye removal experiments in this study were performed as preliminary tests to evaluate the application potential of Cu-HEPES nanosheets beyond phenolic pollutant detection. The observed decrease in UV–vis absorbance indicates dye decolorization/removal under the current conditions. However, the detailed removal mechanism, including the relative contributions of adsorption, catalytic oxidation, and H_2_O_2_-assisted processes, requires further investigation.

## 3. Materials and Methods

### 3.1. Chemicals and Materials

Congo red, copper(II) sulfate pentahydrate, calcium chloride, potassium chloride, sodium carbonate, 4-AAP, zinc acetate dihydrate, HEPES, cadmium chloride, malachite green chloride, TMB, and iron(III) chloride were acquired from Shanghai Adamas Reagent Co., Ltd. (Shanghai, China). 2,4-DCP was acquired from Shanghai Macklin Biochemical Co., Ltd. (Shanghai, China). Magnesium chloride anhydrous was purchased from Chengdu Chron Chemicals Co., Ltd. (Chengdu, China). Sodium chloride was procured from Chengdu Jinshan Chemical Test Co., Ltd. (Chengdu, China). Ethanol absolute was purchased from Chongqing Chuandong Chemical (Group) Co., Ltd. (Chongqing, China). An ultrafiltration centrifuge tube and solid-phase extraction column were acquired from Shanghai Adamas Reagent Co., Ltd. (Shanghai, China).

### 3.2. Instrumentation

The surface morphologies and microstructures of the as-prepared materials were characterized by an SU8100 scanning electron microscope (SEM) (Hitachi, Japan) and observed through transmission electron microscopy (TEM) with a JEM-2100F microscope (JEOL, Tokyo, Japan). X-ray photoelectron spectroscopy (XPS) tests were performed with an Escalab 250Xi system (Thermo Fisher Scientific Inc., Waltham, MA, USA). The pH values during the experiments were monitored with an FE 28 pH meter supplied by Mettler Toledo Technology (China) Co., Ltd. (Shanghai, China), and temperature control was carried out in a DHG-9035A drying oven manufactured by Shanghai Yiheng Technology Instrument Co., Ltd., Shanghai, China. The analysis of Fourier transform infrared (FT-IR) spectra was conducted using a Nicolet iS50 spectrometer (Thermo Fisher Scientific Inc.). The crystal phase composition was analyzed by X’Pert powder X-ray diffraction (XRD) (Empyrean, PANalytical, Almelo, The Netherlands). UV–vis absorption spectra were obtained using a UV-1050 spectrophotometer provided by Techcomp Instrument (Beijing) Co., Ltd. (Beijing, China).

### 3.3. Analysis of the Oxidase-Mimetic and Laccase-Mimetic Activities Exhibited by Cu-HEPES Nanosheets

For assessing the oxidase-mimetic activity of Cu-HEPES nanosheets, 100.0 μL of 80.0 mM CuSO_4_·5H_2_O solution was mixed with 100.0 μL of 100.0 mM, pH 10.0 HEPES solution, resulting in immediate precipitation of Cu-HEPES nanosheets. Unless otherwise stated, Cu-HEPES nanosheets were freshly prepared for each reaction. Specifically, one preparation batch was used entirely as the catalyst for one reaction, thereby ensuring a consistent catalyst dose in all experiments. Each experiment was performed in triplicate under the same conditions. Following centrifugation of the reaction mixture at a rotational speed of 5000 rpm for 1.0 min, the resulting solid sediment was harvested. The activity assay system consisted of 100 μL of 10.0 mM Tris-HCl buffer solution at pH 6.0, 10 μL of 75 μM TMB, and 90 μL of ultrapure water. Following a reaction at 30 °C for 1.0 min, the mixture was centrifuged at 5000 rpm for another 1.0 min to obtain the supernatant. The collected liquid was subsequently subjected to absorbance detection at 458 nm.

Additionally, to determine the laccase-mimetic catalytic performance of Cu-HEPES nanosheets, we freshly prepared a reaction system consisting of 50 μL of 1.67 mM 4-AAP aqueous stock solution, 100 μL of 10.0 mM Tris-HCl buffer solution (pH 6.0), 100 μL of ultrapure water, and 50 μL of 0.042 mM 2,4-DCP solution. Thereafter, Cu-HEPES nanosheets were incorporated into the system, and the formed reaction solution was incubated at 37 °C for 1.0 min, followed by centrifugation at 5000 rpm over the same time span to isolate the supernatant. Finally, the optical absorption value of the collected supernatant was recorded at 500 nm.

### 3.4. Apparent Michaelis–Menten Parameter Analysis of Cu-HEPES Nanosheets

The apparent Michaelis–Menten parameters of Cu-HEPES nanosheets were determined based on fixed-time absorbance responses at different substrate concentrations. For the oxidase-mimetic kinetic assay, TMB was used as the substrate. Freshly prepared Cu-HEPES nanosheets were added to a reaction system containing 10.0 mM Tris-HCl buffer (pH 6.0) and different concentrations of TMB (40–100 μM). The reaction was carried out at 30 °C for 1.0 min. After centrifugation at 5000 rpm for 1.0 min, the absorbance of the supernatant at 458 nm was recorded. The initial reaction rate was calculated from the absorbance change at 458 nm, corresponding to the formation of the oxidized TMB product.

For the laccase-mimetic kinetic assay, 2,4-DCP was used as the substrate and 4-AAP was used as the chromogenic coupling reagent. Briefly, freshly prepared Cu-HEPES nanosheets were added to a reaction system containing 10.0 mM Tris-HCl buffer solution (pH 7.0), 4-AAP (1.67 mM), and different concentrations of 2,4-DCP (0.05–0.15 mM). The reaction was carried out at 30 °C for 1.0 min. After centrifugation at 5000 rpm for 1.0 min, the absorbance of the supernatant at 500 nm was recorded. The initial reaction rate was calculated from the absorbance change at 500 nm, corresponding to the formation of the quinone imine product.

The apparent Michaelis–Menten parameters were obtained by fitting the absorbance responses at different substrate concentrations to the Michaelis–Menten equation: 1/*v* = *K*_m_/(*v*_max_[S]) + 1/*v*_max_. Here, *v* denotes the fixed-time absorbance response under the assay conditions, *K*_m_ stands for the apparent Michaelis–Menten constant, *v*_max_ represents the maximum response, and [S] signifies the substrate concentration.

### 3.5. Detection of 2,4-DCP via the Constructed Colorimetric Technique

Cu-HEPES nanosheets were freshly prepared according to the procedure described in Section 2.3. Subsequently, 100 μL of 10.0 mM Tris-HCl buffer pre-adjusted to pH 6.0, 50 μL of 1.67 mM 4-AAP solution, 50 μL of 2,4-DCP solutions spanning a concentration gradient of 1.67 mM, 3.33 mM, 6.66 mM, 10.0 mM, 13.33 mM, and 16.67 mM, and 100 μL of ultrapure water were added sequentially into the centrifuge tube containing Cu-HEPES nanosheets, followed by incubation at 30 °C for 1.0 min. After centrifuging at 5000 rpm over 1.0 min, the supernatant fraction was isolated and harvested, with its optical absorbance subsequently quantified at a wavelength of 500 nm. All assays were repeated in triplicate to confirm result consistency. Water Sample 1 was tap water collected from Chongqing Medical and Pharmaceutical College (Chongqing, China), while Water Sample 2 was lake water collected from Yunhu Lake at Chongqing University (Chongqing, China). Before analysis, both water samples were filtered through a 0.22 μm membrane filter to remove suspended particulate matter. Subsequently, the filtered sample was spiked with 2,4-DCP at a known concentration, and recovery analysis was performed using the proposed colorimetric method.

### 3.6. Dye Removal

Before the experiment, 100.0 mM H_2_O_2_, 0.2 mM malachite green chloride, and 0.8 mM Congo red were dissolved separately in ultrapure water. The Cu-HEPES nanosheets were combined with 100 μL of ultrapure water, 50 μL of H_2_O_2_ solution (final concentration of 25.0 mM), and 50 μL of either malachite green chloride or Congo red solution to evaluate their removal capacity. Upon incubation of the reaction mixture at 40 °C, the resulting sample was subjected to centrifugation at 5000 rpm over 1.0 min. Next, the supernatant was separated, and spectral detection was performed over a wavelength range of 350–750 nm. Additionally, a control group was implemented with a solution that did not contain Cu-HEPES nanosheets. For the simultaneous dye removal experiment, malachite green and Congo red were used at final concentrations of 0.05 mM and 0.20 mM, respectively. Because malachite green and Congo red have different molar absorptivity and characteristic absorption bands, these concentrations were selected to provide comparable and readily measurable initial absorbance signals at their respective characteristic wavelengths. This allowed both dyes to be clearly tracked in the same UV–vis spectrum and prevented the signal of one dye from masking the spectral change of the other. To prepare this system, 50 µL of 0.2 mM malachite green, 50 µL of 0.8 mM Congo red, 50 µL of H_2_O_2_ solution (final concentration of 25.0 mM), and 50 µL of ultrapure water were added to the Cu-HEPES nanosheets. The dye removal efficiency (%) is determined based on the relative change between the initial absorption peak intensity and the final absorption peak intensity at each time interval:

(1) $$\mathrm{Removal}\;\mathrm{rate}\;(\%)\;=\;(\frac{A_{0}-A_{t}}{A_{0}})\times 100$$

where A_0_ and A_t_ denote the peak absorbance intensities of the dye prior to and following the dye removal process.

## 4. Conclusions

In summary, Cu-HEPES nanosheets with dual enzyme-mimetic activities (oxidase- and laccase-mimetic activities) were synthesized through the coordination of HEPES with copper ions under alkaline conditions. The obtained Cu-HEPES nanosheets were further applied to phenolic pollutant detection and additionally evaluated in preliminary tests of dye decolorization/removal. The synthesized Cu-HEPES nanosheets displayed high dual enzyme-mimetic activities, with a *K*_m_ of 23.2 μM for 2,4-DCP and 0.11 mM for TMB. A colorimetric sensing method developed using this nanozyme achieves a detection limit of 0.3 μM for 2,4-DCP. Finally, the Cu-HEPES nanosheets were applied to preliminarily evaluate their removal performance toward individual and mixed dyes, suggesting their potential for dye decolorization/removal in aqueous systems. Benefiting from the simple synthesis, dual enzyme-mimetic activity, and satisfactory analytical performance, the Cu-HEPES nanosheets show promise as bifunctional nanozymes for rapid colorimetric detection of phenolic pollutants and for preliminary tests of dye decolorization/removal. Nevertheless, several limitations should be acknowledged. The dye removal experiments in this study provide preliminary evidence of dye decolorization/removal based on UV–vis absorbance changes, while complete dye degradation or mineralization was not confirmed. The relative contributions of adsorption, catalytic oxidation, and H_2_O_2_-assisted processes require further systematic investigation using intermediate identification, total organic carbon analysis, radical-trapping experiments, and recycling evaluation. In addition, although satisfactory spike recoveries were obtained in tap water and lake water samples, further validation in more complex real wastewater matrices is needed to assess the practical applicability of the proposed system.

## Figures and Tables

**Table 1:** Comparison of different UV–vis methods for the detection of 2,4-DCP.

Materials	Detection Methods	Linear Range (μM)	LOD (μM)	Ref.
Copper-2-methylimidazole nanosheets	UV–vis	2.5–80	0.9	[21]
Cu_2_O_v_@Ce-tetrakis (4-carboxyphenyl) porphyrin	UV–vis	1.66–183.33	0.72	[24]
Mn-F-Cu sheets	UV–vis	1–100	2.41	[25]
Sr-doped MnO_2_	UV–vis	40–1000	0.992	[26]
Copper ions and 5-(sulfomethyl) isophthalic acid	UV–vis	1–100	0.53	[27]
Copper-based nanoflower	UV–vis	5−100	2.95	[28]
Benzophenone-alanine–Cu	UV–vis	0−40	0.5	[29]
CuCo_2_S_4_	UV–vis	0.5−80	0.39	[30]
Carboxymethylcellulose-Pt nanozyme	UV–vis	6.25−225	0.9	[31]
Cu-HEPES nanosheets	UV–vis	3.3–16.6	0.3	This study

**Table 2:** Detection of 2,4-DCP in real samples using the Cu-HEPES nanosheet-based colorimetric method (*n* = 3).

Samples	Added (μM)	Found ± SD (μM)	Recovery (%)
Water Sample 1	6.6	6.7 ± 1.2	100.2
	10.0	10.7 ± 0.9	106.9
	13.3	13.7 ± 1.3	102.6
Water Sample 2	6.6	6.5 ± 0.9	97.5
	10.0	9.3 ± 0.9	92.7
	13.3	13.3 ± 1.4	99.8

## Data Availability

The original contributions presented in this study are included in the article/Supplementary Materials. Further inquiries can be directed to the corresponding author.

## Supplementary Materials

The following supporting information can be downloaded at: https://www.mdpi.com/article/10.3390/molecules31152639/s1. Figure S1: Apparent Michaelis–Menten parameter analysis of the Cu-HEPES nanosheets with laccase-like activity. The relationship between absorbance responses and 2,4-DCP concentrations (A); Double reciprocal curves of 2,4-DCP catalyzed by the laccase-like activity of Cu-HEPES nanosheets (B). Figure S2: Selectivity (A) and interference (B) studies for the 2,4-DCP detection using the Cu-HEPES nanozyme. Figure S3: Effect of the pH (A), temperature (B), and incubation time (C) on the relative oxidase-like activity of the Cu-HEPES nanosheets. Conditions: temperature = 37 °C, incubation time = 1.0 min for A; pH = 6.0, incubation time = 1.0 min for B; and pH = 6.0, temperature = 30 °C for C. Figure S4: Apparent Michaelis–Menten parameter analysis of the Cu-HEPES nanosheets with oxidase-like activity. The relationship between absorbance responses and TMB concentrations (A); Double reciprocal curves of TMB catalyzed by the oxidase-like activity of Cu-HEPES nanosheets (B). Figure S5: XRD images of the Cu-HEPES nanosheets, congo red, malachite green, congo red incubated with the Cu-HEPES nanosheets, and malachite green incubated with the Cu-HEPES nanosheets. Table S1: Comparison of the Apparent Michaelis–Menten parameters of Cu-HEPES nanozyme and the other reported laccase-like activity nanozymes. Table S2: Comparison of the Apparent Michaelis–Menten parameters of Cu-HEPES nanozyme and the other reported oxidase-like activity nanozymes. Refs. [37,38,39,40,41,42,43,44,45,46,47,48,49,50,51] are cited in the Supplementary Materials.
