# Supplementary material for: Cu-HEPES Nanosheets with Dual Enzyme-Mimetic Activities for Colorimetric Detection of 2,4-Dichlorophenol
Source: Molecules. 2026 Jul 29;31(15):2639. doi: 10.3390/molecules31152639 (PMC13468591; doi:10.3390/molecules31152639)
Supplement: Supplementary file 1 [file molecules-31-02639-s001.zip › molecules-4429255-supplementary.pdf]

Electronic Supplementary Material

Cu-HEPES Nanosheets with Dual Enzyme-Mimetic Activities for Colorimetric Detection of 2,4-Dichlorophenol

Jia-Yuan He [1] and Hao Zhang [2,*]

[1] Traditional Chinese Medicine College, Chongqing University of Chinese Medicine, Chongqing 402760, China; hejiayuan@cqctcm.edu.cn
[2] Chongqing Engineering Research Center of Pharmaceutical Sciences, Chongqing Medical and Pharmaceutical College, Chongqing 401331, China
* Correspondence: 2110011@cqmpc.edu.cn

Table of contents

Figure S1. Apparent Michaelis-Menten parameter analysis of the Cu-HEPES nanosheets with laccase-like activity. The relationship between absorbance responses and 2,4-DCP concentrations (**A**); Double-reciprocal curves of 2,4-DCP catalyzed by the laccase-like activity of Cu-HEPES nanosheets (**B**).

Figure S2. Selectivity (**A**) and interference (**B**) studies for the 2,4-DCP detection using the Cu-HEPES nanozyme.

Figure S3. Effect of the pH (**A**), temperature (**B**), and incubation time (**C**) on the

relative oxidase-like activity of the Cu-HEPES nanosheets. Conditions: temperature = 37°C, incubation time = 1.0 min for **A**; pH = 6.0, incubation time = 1.0 min for **B**; and pH = 6.0, temperature = 30°C for **C**.

Figure S4. Apparent Michaelis-Menten parameter analysis of the Cu-HEPES nanosheets with oxidase-like activity. The relationship between absorbance responses and TMB concentrations (**A**); Double-reciprocal curves of TMB catalyzed by the oxidase-like activity of Cu-HEPES nanosheets (**B**).

Figure S5. XRD images of the Cu-HEPES nanosheets, congo red, malachite green, congo red incubated with the Cu-HEPES nanosheets, and malachite green incubated with the Cu-HEPES nanosheets.

Table S1 Comparison of the Apparent Michaelis-Menten parameters of Cu-HEPES nanozyme and the other reported laccase-like activity nanozymes.

Table S2 Comparison of the Apparent Michaelis-Menten parameters of Cu-HEPES nanozyme and the other reported oxidase-like activity nanozymes.

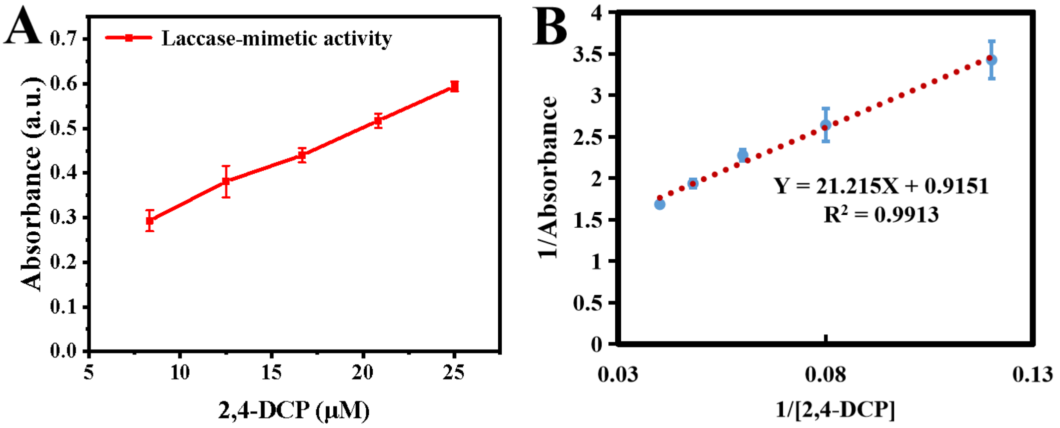

Figure S1. Apparent Michaelis-Menten parameter analysis of the Cu-HEPES

nanosheets with laccase-like activity. The relationship between absorbance responses and 2,4-DCP concentrations (**A**); Double-reciprocal curves of 2,4-DCP catalyzed by the laccase-like activity of Cu-HEPES nanosheets (**B**).

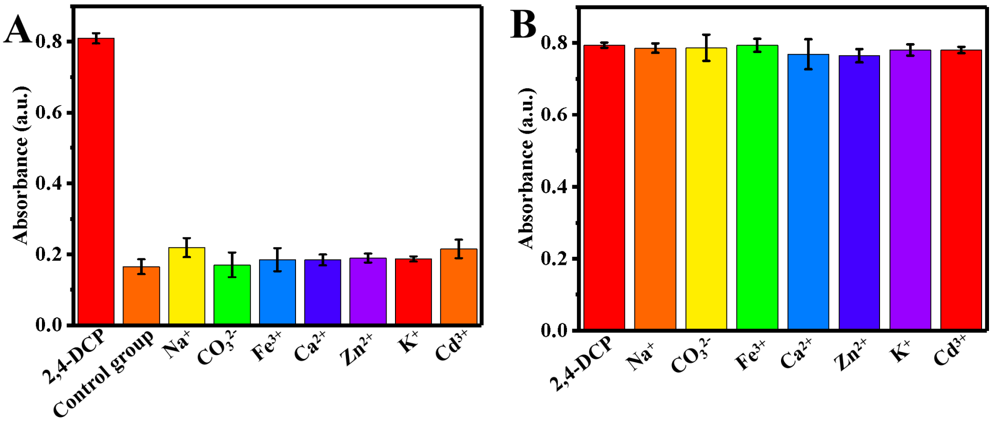

Figure S2. Selectivity (**A**) and interference (**B**) studies for the 2,4-DCP detection using the Cu-HEPES nanozyme.

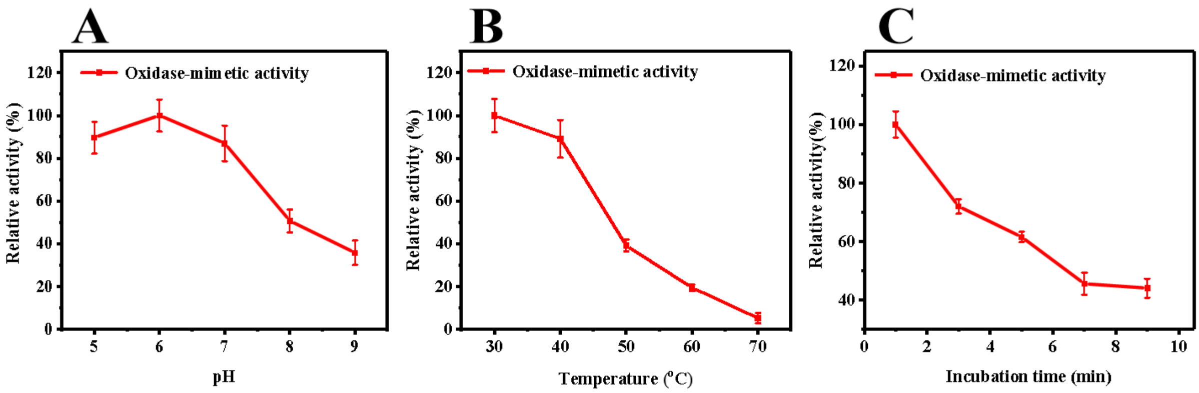

Figure S3. Effect of the pH (**A**), temperature (**B**), and incubation time (**C**) on the relative oxidase-like activity of the Cu-HEPES nanosheets. Conditions: temperature = 37°C, incubation time = 1.0 min for **A**; pH = 6.0, incubation time = 1.0 min for **B**; and pH = 6.0, temperature = 30°C for **C**.

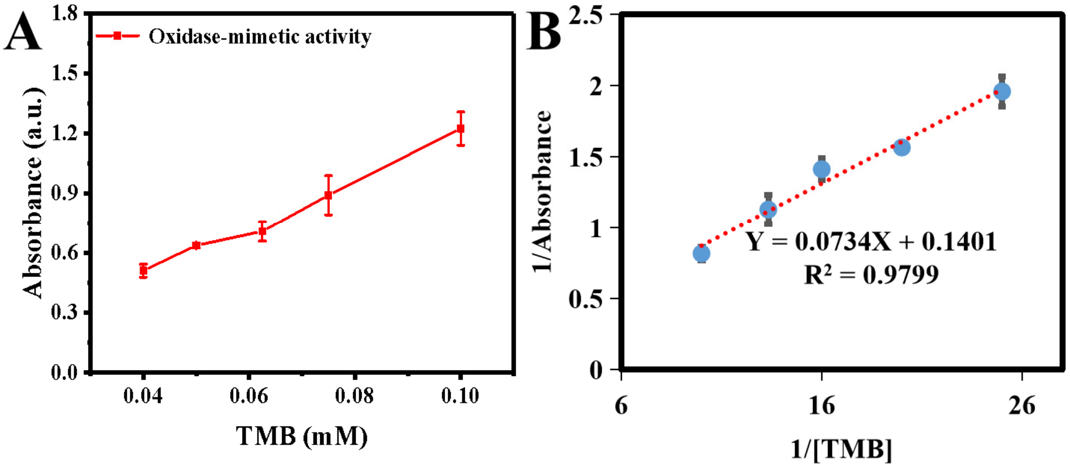

Figure S4. Apparent Michaelis-Menten parameter analysis of the Cu-HEPES nanosheets with oxidase-like activity. The relationship between absorbance responses and TMB concentrations (**A**); Double-reciprocal curves of TMB catalyzed by the oxidase-like activity of Cu-HEPES nanosheets (**B**).

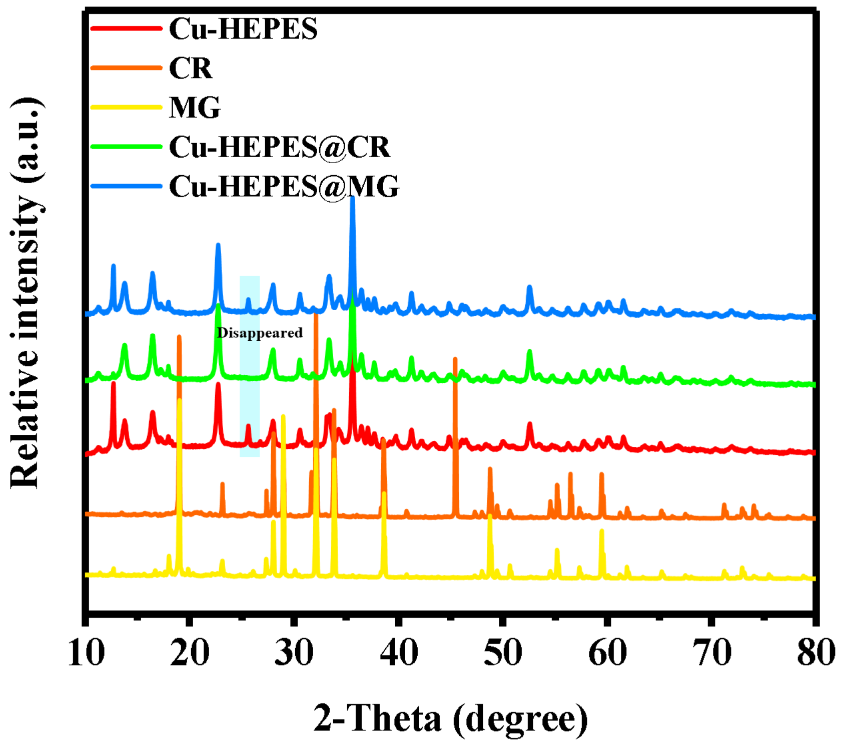

Figure S5. XRD images of the Cu-HEPES nanosheets, congo red, malachite green, congo red incubated with the Cu-HEPES nanosheets, and malachite green incubated with the Cu-HEPES nanosheets.

Table S1 Comparison of the Apparent Michaelis-Menten parameters of Cu-HEPES nanozyme and the other reported laccase-like activity nanozymes.

**Nanozymes with laccase-like activity**	K_m **(mM)**	**Ref.**
Cu_2O_v@Ce-TCPP	7.5	[24]
Cu-MN	0.05	[28]
CMC-Pt NPs	0.218	[31]
Laccase	0.094	[32]
Cu_2O-I	0.147	[32]
AP-Cu	0.14	[37]
CA-Cu	0.232	[38]
CuMn aminoclays-1–7	0.63	[39]

Tris-Cu	0.18	[40]
Cu-ATZ	0.08	[41]
Cu-HEPES	0.023	This study

TCPP: tetrakis (4-carboxyphenyl) porphyrin; Cu-MN: Complex of 2-aminoterephthalic acid and 2-methylimidazole with copper; CMC-Pt NPs: carboxymethylcellulose-platinum nanoparticles; AP: adenine phosphate; CA-Cu: Complex of cyanuric acid (CA) with Cu^{2+}; Cu-ATZ: Complex of 2-amino-1,3,4-thiadiazole (ATZ) with copper.

Table S2 Comparison of the Apparent Michaelis-Menten parameters of Cu-HEPES nanozyme and the other reported oxidase-like activity nanozymes.

**Nanozymes with oxidase-like activity**	**K_m (mM)**	**Ref.**
N, C co-doped Co_3O_4	0.13	[42]
PEI@Mn/Cu^{2+}-CDs	0.415	[43]
Co_3O_4@MnO_2	0.198	[44]
Fe/Mn-ZIF	0.39	[45]
Mn–N–C SAzymes	0.12	[46]
SapYZUM13@Mn_3O_4-NH_2	0.49	[47]
Mn-CeO_2	0.25	[48]
FeS_2–MoS_2	0.488	[49]

Fe_3O_4@o-PPy NPs	11.86	[50]
Iron-doped nitride-rich carbon	0.363	[51]
Cu-HEPES	0.52	This study

PEI: polyethyleneimine; CDs: carbon dots; ZIF: Zeolitic imidazolate framework; SAzymes: single-atom nanozymes; Fe_3O_4@o-PPy NPs: Fe_3O_4@oxygen-functionalized polypyrrole nanoparticles.
